# Supplementary material for: Transcriptome Analysis Reveals Vimentin-Induced Disruption of Cell–Cell Associations Augments Breast Cancer Cell Migration
Source: Cells. 2022 Dec 13;11(24):4035. doi: 10.3390/cells11244035 (PMC9776984; doi:10.3390/cells11244035)
Supplement: Supplementary file 1 [file cells-11-04035-s001.zip › DEGs and enrichment analysis.zip!/DEGs and enrichment analysis/WT_VIMvsVector GO enrichment/ALL/WT_VIMvsVector_all.GObp_DAG.pdf]

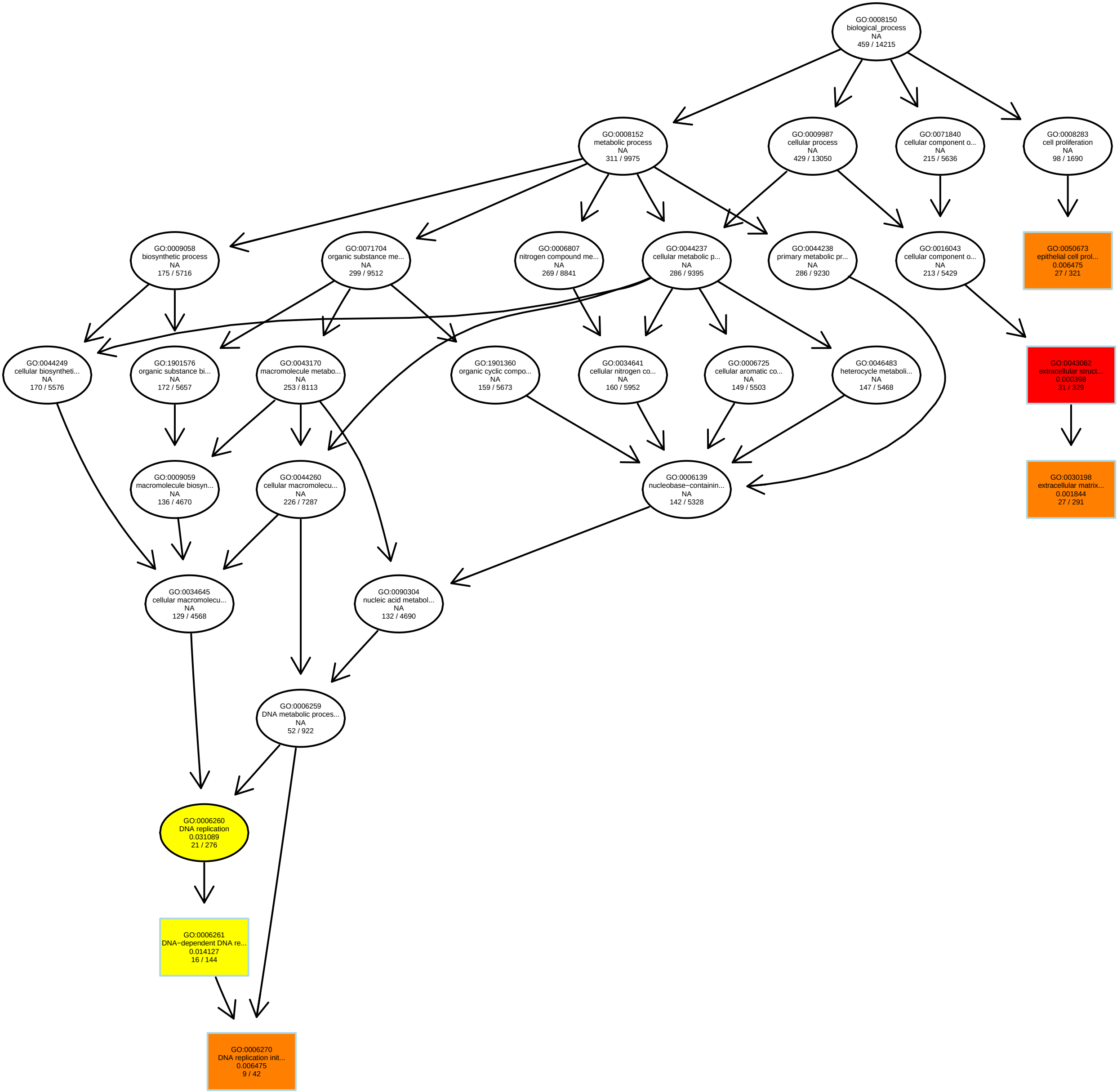
GO:0008150
biological_process
NA
459 / 14215
GO:0008152
metabolic process
NA
311 / 9975
GO:0009987
cellular process
NA
429 / 13050
GO:0071840
cellular component o...
NA
215 / 5636
GO:0008283
cell proliferation
NA
98 / 1690
GO:0009058
biosynthetic process
NA
175 / 5716
GO:0071704
organic substance me...
NA
299 / 9512
GO:0006807
nitrogen compound me...
NA
269 / 8841
GO:0044237
cellular metabolic p...
NA
286 / 9395
GO:0044238
primary metabolic pr...
NA
286 / 9230
GO:0016043
cellular component o...
NA
213 / 5429
GO:0050673
epithelial cell prol...
0.006475
27 / 321
GO:0044249
cellular biosyntheti...
NA
170 / 5576
GO:1901576
organic substance bi...
NA
172 / 5657
GO:0043170
macromolecule metabo...
NA
253 / 8113
GO:1901360
organic cyclic compo...
NA
159 / 5673
GO:0034641
cellular nitrogen co...
NA
160 / 5952
GO:0006725
cellular aromatic co...
NA
149 / 5503
GO:0046483
heterocycle metaboli...
NA
147 / 5468
GO:0043062
extracellular struct...
0.000398
31 / 329
GO:0009059
macromolecule biosyn...
NA
136 / 4670
GO:0044260
cellular macromolecu...
NA
226 / 7287
GO:0006139
nucleobase−containin...
NA
142 / 5328
GO:0030198
extracellular matrix...
0.001844
27 / 291
GO:0034645
cellular macromolecu...
NA
129 / 4568
GO:0090304
nucleic acid metabol...
NA
132 / 4690
GO:0006259
DNA metabolic proces...
NA
52 / 922
GO:0006260
DNA replication
0.031089
21 / 276
GO:0006261
DNA−dependent DNA re...
0.014127
16 / 144
GO:0006270
DNA replication init...
0.006475
9 / 42
